# Treatment of chronic osteomyelitis with gradient release of DGEA and vancomycin hydrogel-microsphere system and its mechanism

**DOI:** 10.3389/fphar.2024.1499742

**Published:** 2024-11-11

**Authors:** Yuhao Zheng, Yue Wang, Fan Sheng, Shu Wang, Ying Zhou, Xiaoxu Li, Ning Li, Wenling Song, Zhiming Song

**Affiliations:** ^1^ Department of Sports Medicine, Orthopedics’ Clinic, The First Hospital of Jilin University, Changchun, Jilin, China; ^2^ Department of Dermatology, The Second Hospital of Jilin University, Changchun, Jilin, China; ^3^ Department of Radiotherapy, The Second Hospital of Jilin University, Changchun, Jilin, China; ^4^ Department of Obstetrics, The First Hospital of Jilin University, Changchun, Jilin, China

**Keywords:** thermo-sensitive hydrogel, microsphere, sequential drug release, osteomyelitis, drug delivery

## Abstract

In recent years, the treatment of chronic osteomyelitis mediated by biodegradable polymer platforms has received increasing attention. This paper reports an advanced drug delivery system, vancomycin (VA) and DGEA loaded microspheres embedded in injectable thermosensitive polypeptide hydrogels (i.e., hydrogel-microsphere (Gel-MP) construct), for continuous release of drugs with different mechanisms and more comprehensive treatment of chronic osteomyelitis. The Gel-MP construct exhibits continuous biodegradability and excellent biocompatibility. Microspheres (MP) are wrapped inside Gel. With the degradation of Gel, VA and MP are released from them, VA released with faster degradation speed, achieving a potent antibacterial effect and effectively controlling infection. Due to the slower degradation rate of MP compared to Gel, subsequently, DGEA is released from MP to induce bone formation and produce the effect of filling bone defects. Compared with other formulations, the *in vivo* combinational treatment of Gel/VA-MP/DGEA can simultaneously balance antibacterial and osteogenic effects. More importantly, local sustained-release drug delivery systems can significantly mitigate the systemic toxicity of drugs. Therefore, the injection local sequential drug delivery system has broad prospects in the clinical application of treating chronic osteomyelitis.

## 1 Introduction

Chronic osteomyelitis is an inflammatory disease caused by pathogen infection, primarily impacting that affects the periosteum and bone cortex, accompanied by bone destruction, often leading to dead bone and the formation of localized bone intima (Bury et al., 2021). Chronic osteomyelitis has always been an urgent clinical challenge due to its long course, high recurrence rate, and antibiotic resistance. The treatment of chronic osteomyelitis in clinical practice primarily encompasses targeted antibacterial therapy, surgical debridement, and addressing internal complications (Urish and Cassat, 2020). Due to the uncertainty of the time threshold for acute osteomyelitis to develop into chronic osteomyelitis, recurring episodes often result in failed clinical interventions. This has profound implications for patient quality of life and imposes a substantial burden on healthcare systems’ costs (Fantoni et al., 2019). Chronic osteomyelitis is often accompanied by necrotic tissue without blood circulation and bacterial biofilms that hinder antibiotic penetration. Generally, monotherapy with antibiotics is difficult to achieve cure. The treatment of chronic traumatic osteomyelitis usually involves thorough debridement combined with the use of antibiotics. Non intestinal antibiotic treatment for 4–6 weeks has become the standard treatment protocol for chronic osteomyelitis. Long term systemic high-dose infusion of antibiotics frequently leads to nephrotoxicity, ototoxicity, and severe gastrointestinal reactions (Patangia et al., 2022), which encourages researchers to develop effective strategies to mitigate these issues.

In recent years, many studies have focused on *in situ* drug delivery systems. Effective local antibiotic treatment after surgical debridement has the characteristics of targeted slow-release to the lesion site, replacing systemic antibiotics, reducing drug toxicity and side effects, and increasing local drug concentration. Both domestically and internationally, PMMA beads are utilized in the treatment of chronic osteomyelitis, but the bead chain needs to be removed in a second surgery, which brings inconvenience to patients and has no osteogenic effect, making it impossible to repair bone defects (Liu et al., 2022). Calcium phosphate bone cement (CPC) is a synthetic bone substitute material made from self-curing non ceramic hydroxyapatite (HAP) and has recently received considerable attention in clinical practice. Drug loaded CPC has received numerous clinical trials due to its ability to extend the duration of drug efficacy, possess good antibacterial effects, biocompatibility, and bone induction ability (Lang et al., 2021). However, bone cement has problems such as high brittleness, poor anti erosion performance, limited drug loading, impact on antibiotic release after solidification, and a brief duration of antibiotic release (Wong et al., 2021). This not only prolongs the treatment time of patients, but also increases their economic burden. Therefore, finding a local drug delivery system that can effectively release antibiotics for a long time and degrade them has always been a research goal for clinical workers.

Hydrogel is a kind of macromolecular polymer with three-dimensional network structure that can be swelled in water and maintain a certain amount of water, while it is insoluble (Cao et al., 2021). Injectable thermosensitive hydrogel can encapsulate therapeutic drugs or cells by simple mixing, act as drug release repository or growth scaffold *in vivo*, and can be self-degraded and cleared after release (Shi et al., 2021). Vancomycin (VA) is the most commonly used and effective antibiotic in the treatment of chronic osteomyelitis, demonstrating substantial antibacterial effects against osteomyelitis caused by a wide range of bacteria (Aggarwal et al., 2022; Cai et al., 2021). Compared with systemic administration, the local continuous release of drugs from the Gel at the osteomyelitis site can effectively enhance treatment efficacy (Zhao et al., 2019), and its physical properties such as porosity can be designed according to the drug demand. While providing a large drug load, it provides high surface volume ratio, high water retention performance, biocompatibility, and reduces the systemic toxicity of drugs, Therefore, it is a feasible way to choose hydrogel to carry macromolecular drugs to achieve controllable release (Pandey et al., 2016).

Microspheres (MPs) are widely used in drug controlled delivery (García-González et al., 2015). Currently, various polymers have been used to prepare MPs. Among them, the biodegradable synthetic polymer poly (lactic acid glycolic acid) (PLGA) has received widespread attention (Kontakis et al., 2007; Yoo and Won, 2020; Zhang P. et al., 2023). Previous studies have shown that MPs made from PLGA can encapsulate many small molecule drugs for the treatment of chronic osteomyelitis, such as vancomycin (VA), hydroxyapatite, levofloxacin, bone morphogenetic protein-2 (BMP-2) (Wang et al., 2023; Kuang et al., 2021; Zhan et al., 2024; Guo et al., 2020), etc., With the gradual degradation of PLGA skeleton, microspheres loaded with small molecule drugs can stably encapsulate and release drugs, achieving long-term sustained drug delivery. DGEA is a newly discovered peptide derived from type I collagen, which is the main component of organic bone matrix. Type I collagen binding and activation α2β1 Integrated protein (Leung, 2004), while α2β1 is a receptor that can induce osteogenic differentiation of bone progenitor cells. Therefore, DGEA has a good osteogenic induction effect and promotes osteogenesis (Bi et al., 2018; Mehta et al., 2015). With the rapid development of transportation and the aggravation of hospital infections (orthopedic fixation equipment and total joint implants) in recent years, there are more and more patients with bone fractures and osteomyelitis. These patients often have large irregular bone defects, slow bone repair, and are not easy to grow. They even produce open sinusoids, exacerbating soft tissue defects and bone defects (Wiese and Pape, 2010; Lima et al., 2014). The formation of bone defects in chronic osteomyelitis is a difficult treatment point. Applying DGEA to bone defects caused by chronic osteomyelitis would be a feasible supplement to the treatment system for chronic osteomyelitis.

**SCHEME 1:**
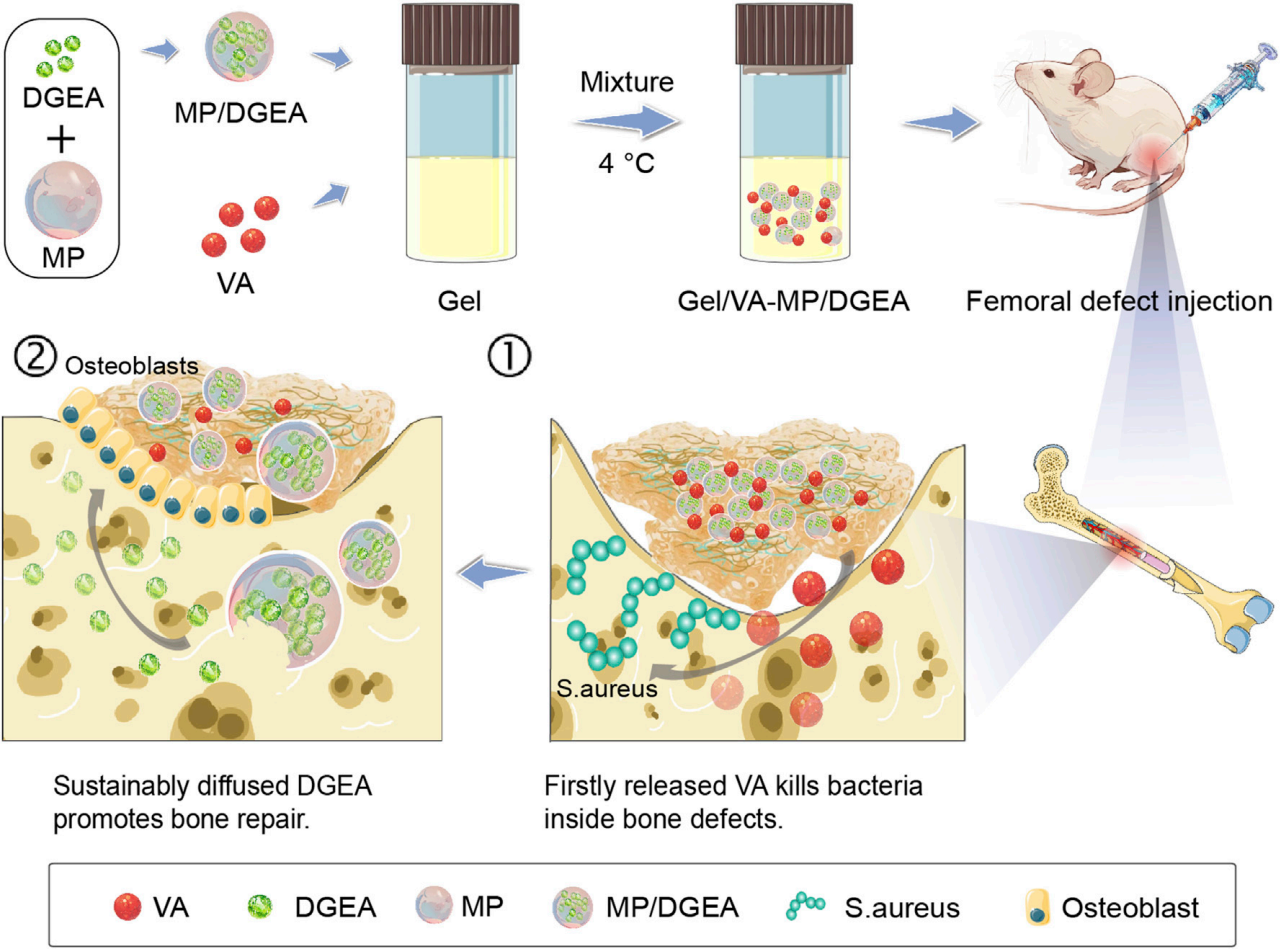
The sequential administration of Gel-MP structure demonstrates the preparation of Gel/VA-MP/DGEA for the treatment of chronic osteomyelitis. The MP were loaded with DGEA, and the Gel were loaded with VA. It can be injected into the defect area of osteomyelitis. Gel/VA is released first to control infection, and then MP/DGEA is released to promote the healing of osteomyelitis.

In addressing the aforementioned challenges in this study, a gradient release DGEA and vancomycin (VA) microspheres/hydrogel system was prepared for the treatment of osteomyelitis in bone defects. However, Literature regarding its application in the treatment of osteomyelitis and bone defects is relatively scarce, and there is a lack of in-depth bacterial and animal *in vivo* experiments. As shown in the Scheme 1, the composite microspheres are encapsulated within the Gel. First, the Gel degrades, and VA encapsulated in the hydrogel is released preferentially to play an antibacterial role in the treatment of osteomyelitis. Subsequently, the microspheres gradually degrade, and the sustained release of DGEA induces bone formation in the following 4 months, repairing bone defects caused by chronic osteomyelitis. Our work shows that hydrogel-microspheres (Gel-MP) have good application prospects in the construction of chronic osteomyelitis.

## 2 Materials and methods

### 2.1 Materials

All materials used in this work have been explained in the Supplementary Material.

### 2.2 Fabrication and characterizations of Gel-MP construct

#### 2.2.1 Preparations and characterizations of Gel and MP

The composition of Gel is the copolymer of poly (L-alanine-*co*-L-phenylalanine)-*block*-poly (ethyleneglycol)-*co*-poly (L-alanine-*co*-L-phenylalanine) (PLAF-*b*-PEG-*b*-PLAF). As depicted in Supplementary Scheme S1, the copolymer of PLAF-*b*-PEG-*b*-PLAF was synthesized through the ring-opening polymerization (ROP) of L-alanine N-carboxyanhydride (L-Ala NCA) and L-phenylalanine *N*-carboxyanhydride (L-Phe NCA) using amino-terminated poly-(ethylene glycol) (NH2-PEG-NH2) as a macroinitiator. The blank and DGEA-loaded MPs were prepared by oil in water (W1/O/W2) dual lotion technology. Detailed procedures for the preparation and characterization of Gel-MP are described in the Supplementary Material.

#### 2.2.2 Preparation of drug-loaded Gel-MP

The main components of MP is Poly (lactide-co-glycolide) (PLGA). The concentration of DGEA embedded in MP is determined based on its drug loading capacity (DLC) (9.88 wt%) and the required dose of 15.0 mg per kg body weight for osteogenesis (mg (kg BW)^−1^. The concentration of VA is determined based on the dosage of 25.0 mg (kg BW)^−1^ VA. For the preparation of Gel/VA-MP/DGEA, 8.0 wt% PLAF-*b*-PEG-*b*-PLAF with 3.9 wt% MP/DGEA and 0.5 wt% VA was dissolved together in phosphate-buffered saline (PBS). Stir the mixed system at 4°C for 3 days to obtain a uniform solution. Gel/VA-MP and Gel-MP/DGEA were prepared by dispersing different components in the same proportion.

#### 2.2.3 *In vitro* degradation and release experiment of Gel-MP

0.5 mL of Gel/VA-MP/DGEA was put into a vial with an inner diameter of 11 mm and placed in a 37°C incubator. After 30 min, the Gel was formed. The quality of Gel/VA-MP/DGEA was weighed with an electronic scale and recorded. Then PBS solution containing or without 2.0 mg mL^−1^ elastase was added into the vial. This is to simulate the degradation microenvironment *in vivo*. When PBS was added with a pipette gun, the liquid should be gently injected along the bottle wall to the upper layer of Gel. Then place it in a 37°C constant temperature chamber and oscillate at 70 rpm min^−1^. Every other day, suck out the upper buffer solution and put it into a 2 mL centrifuge tube to measure the drug release amount of the sample, dry the residual PBS on the bottle wall with filter paper strips, and weigh the Gel. Then add new buffer solution again until the Gel is completely degraded. Collect the quality change of Gel/VA-MP/DGEA to show the degradation process of Gel. Use high-performance liquid chromatography to measure the concentration of drugs in the reserved degradation solution. After exploration, acetonitrile: water (60:40, v/v) is used as the mobile phase, the flow rate is 1.0 mL min^−1^, the absorption peak is set at 230 nm, the absorption peak of DGEA appears at 6 min, and the absorption peak of VA is 6.5 min.

#### 2.2.4 *In vivo* degradation and biocompatibility test of Gel-MP

Sprague Dawley (SD) rats were used in the experiment of hydrogel degradation *in vivo* and biocompatibility. Dissolve the poly (amino acid) polymer in PBS buffer solution and prepare an 8 wt% polymer solution containing 5.0 wt% MP. Stir and dissolve at a constant temperature for 3 days in a refrigerator at 4°C. Inject 500 µL of polyamino acid polymer solution subcutaneously into the back of SD rats using a syringe. The rats were killed at different set times (30 min, 14 days, 28 days, 42 days), the skin of the Gel was cut off, and the degradation of the Gel *in vivo* was observed. At the same time, the skin of the Gel attachment was cut with scissors, stored in 4.0% paraformaldehyde solution, embedded in paraffin, made into 5 μm sections, stained with H&E, and studied the histological changes of the skin under the optical microscope.

### 2.3 *In vitro* antibacterial properties of Gel/VA-MP/DGEA

#### 2.3.1 Bacterial coating experiment

To evaluate the inhibitory effects of Gel/VA-MP/DGEA on *S. aureus* and *E. coli*, 1 g/100 mL of Tryptone, 0.5 g/100 mL of yeast extract, 1 g/100 mL of sodium chloride, and 1.5 g/100 mL of agar powder were taken. The pH of the culture medium was adjusted to 7.4 using NaOH, and the plate was inverted after steam sterilization under high pressure. Take 1 mL of the bacterial solution from *S. aureus* and *E. coli*, add it to a shaking tube containing 4 mL of LB liquid culture medium, and shake well overnight in the shaking bed. Take a solid culture medium plate for coating, with a bacterial liquid volume of 100 μL. Three groups of composite materials were processed into regular circular discs with a diameter of 6 mm and a weight of 2 mg using a 6 mm punch. Two discs were used in each group for the *S. aureus* and *E. coli* coating experiments. Place three group material discs in clockwise order in each culture dish. Take photos separately at 24 h, 3 and 7 days.

#### 2.3.2 Bacterial shaking experiment

Take 100 mL of shaking tubes containing *S. aureus* and *E. coli* respectively, and place them into a Tecan enzyme-linked immunosorbent assay (ELISA)reader to measure absorbance. Calculate the OD value at 625 nm and the dilution factor to approximately 0.05 OD. Take 5 mL of EP tube and calculate the total amount of bacterial solution and LB liquid culture medium according to the dilution ratio, totaling 4 mL. Add each group of materials to the EP tube. Place the bacteria in a 37°C constant temperature shaking bed for 100 r/min, and measure the absorbance using a UV absorbance meter Tecan after 2, 4, 8, 12, and 24 h. Perform statistical analysis after comparing with the blank group.

### 2.4 Femoral osteomyelitis mouse model

#### 2.4.1 Animal procedures

Male SD rats, 6 in each group, totaling 48, weighing 250–350 g, provided by the School of Basic Medicine of Jilin University. All animal experiments are conducted in accordance with animal testing standards and approved by the Animal Experiment Ethics Committee of the First Hospital of Jilin University. Rats inhale isoflurane for anesthesia, and the surgical area is disinfected with iodine. Insert a 0.5 mm sterile surgical drill bit into the bone marrow cavity in the upper third of the rat femur, and rinse the bone marrow cavity with physiological saline. Micro syringe injection 10^2^ CFU (colony forming unit) *S. aureus* (*Staphylococcus aureus*) bacterial solution (Supplementary Figure S1). Suture the skin and muscles of the rats one by one and disinfect them with iodine. Throughout the entire experiment, no antibiotics were injected into the animals.

#### 2.4.2 *In vivo* treatment efficacy

After successful animal modeling, a cotton swab was dipped into the bone marrow cavity blood at the modeling site to conduct a bacterial plate coating experiment. Microbial growth was observed, indicating successful modeling in each group (Supplementary Figure S2). One week after modeling chronic osteomyelitis of the femur in rats, the skin and muscle layers were cut open to expose the femur. Open a rectangular window (5 mm × 1.5 mm) at the flat part of the upper third of the femur, perform debridement according to the normal surgical procedure, and inject different groups of materials into the bone marrow cavity according to the grouping method at the bone defect site 100.0 μL, and fill the bone defect area with the material, the injection method is shown in Supplementary Figure S3. The formulations were abbreviated as follows:

• Group 1: PBS as control group (control);
• Group 2: blank MP-loaded Gel group (Gel-MP);
• Group 3: group of 5.0 mg mL^−1^ free VA in PBS (VA);
• Group 4: group of 3.0 mg mL^−1^ free DGEA in PBS (DGEA);
• Group 5: group of free VA and free DGEA at a concentration of 5.0 and 3.0 mg mL^−1^ in PBS, respectively (VA + DGEA);
• Group 6: VA and blank MP-loaded Gel group (Gel/VA-MP);
• Group 7: group of Gel with DGEA-loaded MP (Gel-MP/DGEA);
• Group 8: VA and MP/DGEA coloaded Gel (Gel/VA-MP/DGEA).

Suture the skin and muscles of the rats one by one to complete the surgery. Afterwards, the rats were raised normally, and their repair status was observed during the feeding period. After being euthanized for 4 months, gross anatomical photos were taken for relevant experiments to evaluate the effectiveness of composite materials in treating chronic osteomyelitis.

### 2.5 *In vivo* therapeutic effects

#### 2.5.1 External phase of femur and Micro-CT analysis

This study used the external appearance of the femur and Micro-CT to detect the target area of the specimen and analyzing the healing status of osteomyelitis after treatment. Observe the external appearance of the femur after dissecting rats 4 months later, fix the sample and place it in the Micro-CT instrument; Then slice and perform spiral scanning with a resolution of 10 μm. Obtain X-ray images by using Imalytics preclinical 2.1 software, subsequently synthesizing and reconstructing the three-dimensional morphology of the tissue. Finally, analyze the bone volume fraction (BV/TV).

#### 2.5.2 Histological and immunohistochemical analysis

After dissecting the complete femur, specific infected bone tissue from the upper third of the femur was taken for HE and Masson staining. The collected femur was fixed overnight in 4% (W/V) PBS, buffered with paraformaldehyde, and then embedded in paraffin. Antigen repair was performed on the paraffin embedded tissue first. HE and Masson staining of paraffin embedded femoral sections for ∼5 μm, slice ∼3 μm for immunohistochemical analysis. Immunohistochemical staining was performed according to the previously reported immunocytochemical method (Zheng et al., 2017). Histological and immunohistochemical changes were detected using a microscope (Nikon, Model Eclipse Ti. Optical Instruments, Admore, Pennsylvania), and then analyzed using ImageJ software (National Institutes of Health, Bethesda, Maryland).

#### 2.5.3 Real-time quantitative polymerase chain reaction (RT-qPCR)

Eight groups of rats were euthanized for 4 months and their expression of bone differentiation genes (OPN, BSP, COL1A1, BMP2, GAPDH) was detected by using RT-qPCR. Cells were lysed with TRIZOL reagent to extract RNA precipitates. The complementary DNA (cDNA) was synthesized using the PrimeScript™ RT Master Mix (Perfect Real Time) reverse transcription kit (Takara, Japan), and gene expression was determined after amplification. The primer sequences used are listed in Table 1. After dissecting the complete femur, the bone defect tissue was taken for RT-PCR to detect the expression of bone differentiation genes (OPN, BSP, COL1A1, BMP2, GAPDH).

**TABLE 1:** Primers sequences used in RT-qPCR.

Gene	Forward primer (5′to 3′)	Reverse primer (5′ to 3′)
OPN	CCA​GCC​AAG​GAC​CAA​CTA​CA	AGT​GTT​TGC​TGT​AAT​GCG​CC
BSP	CCG​GCC​ACG​CTA​CTT​TCT​T	TGG​ACT​GGA​AAC​CGT​TTC​AGA
COL1A1	TGA​CTG​GAA​GAG​CGG​AGA​GT	GAA​TCC​ATC​GGT​CAT​GCT​CT
BMP2	TAG​TGA​CTT​TTG​GCC​ACG​ACG	GCT​TCC​GCT​GTT​TGT​GTT​TG
GAPDH	AGA​CAG​CCG​CAT​CTT​CTT​GT	CTT​GCC​GTG​GGT​AGA​GTC​AT
	TAC​AAC​CTC​CTT​GCA​GCT​CC	GGA​TCT​TCT​GAG​GTA​GTC​AGT​C

#### 2.5.4 Western blot (WB) analysis

Eight groups of rats were euthanized for 4 months and Western blot was used to detect the expression of bone differentiation related proteins (OPN, BSP, COL1A1, BMP2, GAPDH) in each component. The total protein was extracted from cells of each group using radio immunoprecipitation assay, and the protein content was determined. The total protein was transferred to a polyvinylidene fluoride membrane. After sealing, incubating antibodies, and developing them, the grey value was measured using ImageJ software, and the protein expression was quantitatively analyzed.

## 3 Results and discussion

### 3.1 Preparation and characterization of Injectable Gel-MP construct

**FIGURE 1:**
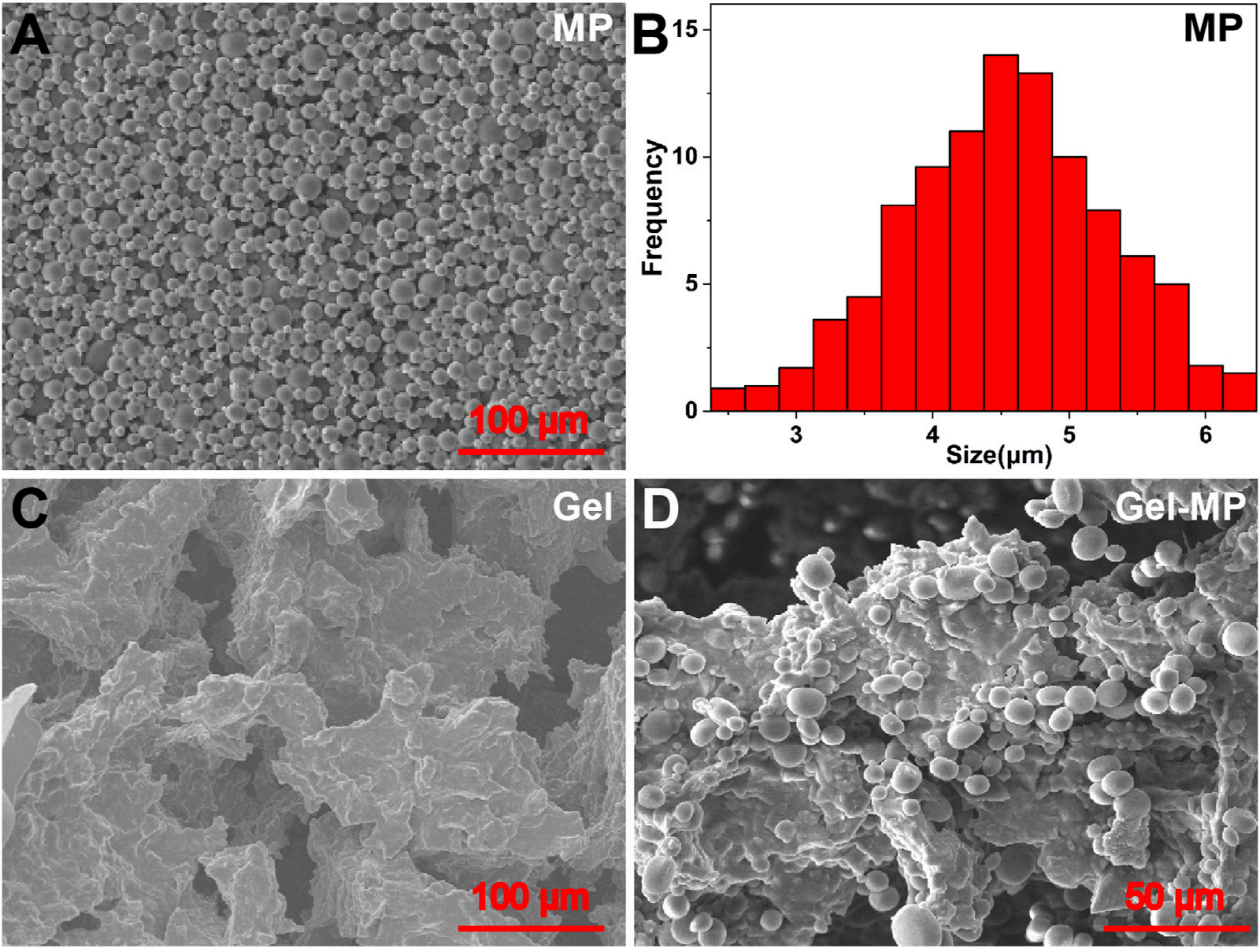
Morphologies and scales of MP, Gel, and Gel-MP: **(A)** SEM micro-image of MP, **(B)** average size of MP, **(C)** SEM micro-images of Gel, and **(D)** Gel-MP.

As shown in Supplementary Scheme S1, PLAF-*b*-PEG-*b*-PLAF was synthesized through the ROP of L-Ala NCA and L-Phe NCA in the presence of NH_2_-PEG-NH_2_, and its chemical structure was characterized by proton nuclear magnetic resonance (^1^H NMR) spectroscopy (Supplementary Figure S4). The degree of polymerization (DPs) of L-Ala and L-Phe units were analyzed by integrating the peaks at 4.67 and 4.41 ppm relative to the peak at 3.67 ppm, with values of 30 and 4, respectively. The number average molecular weight (M_n_) and molecular weight distribution (PDI) of 6,720 g mol^−1^ and 1.47 were determined by ^1^H NMR and Gel permeation chromatography (GPC), respectively. PLGA MP was prepared by W1/O/W2 double emulsion technique. Scanning electron microscopy (SEM) images show that MP exhibits excellent spheroidization and smooth surface (Figure 1A), with an average size of ∼4.53 μm (Figure 1B). DGEA can be efficiently encapsulated within the MP, achieving a DLC of 9.88 wt% and a drug loading efficiency (DLE) of 98.8 wt%. Therefore, PLGA MP is deemed a promising carrier for the encapsulation and delivery of DGEA.

**FIGURE 2:**
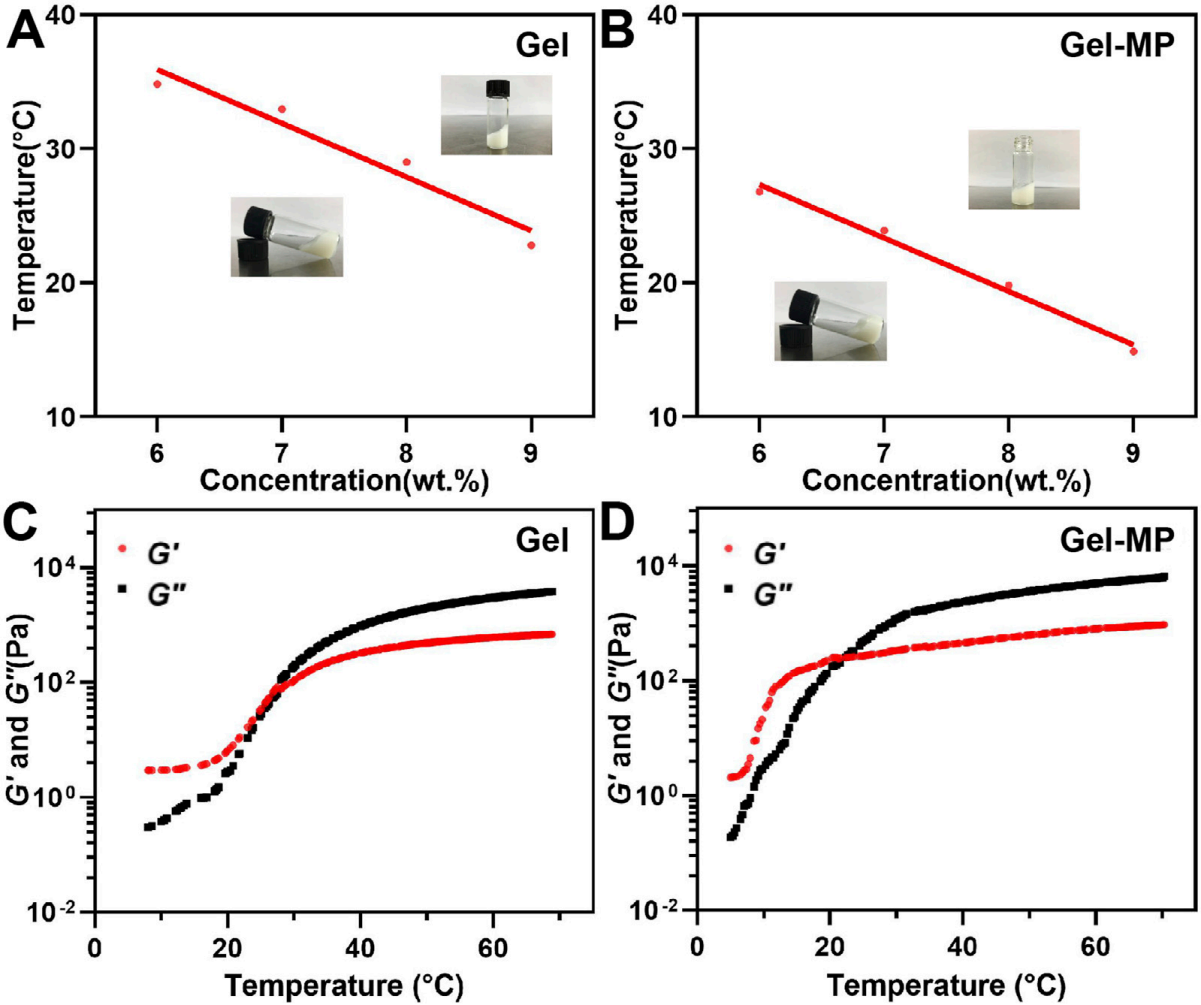
Phase diagrams and rheological properties. **(A)** Gel phase diagram with broad photographs before and after gel formation. **(B)** Gel-MP phase diagram with broad photographs before and after gel formation. **(C)** G′ (Storage modulus) and G″ (Loss modulus) of Gel. **(D)** G′ and G″ of Gel-MP.

The phase diagram determines the gel formation temperature of various gel concentrations based on the tilt of the vial, as shown in Figures 2A, B. When the vial is tilted, if the liquid level flows, the solution is in the Sol-Gel state; if the liquid level does not flow, the solution is in the gel state. With the increase of the temperature, the PLAF-*b*-PEG-*b*-PLAF solution experiences a Sol-to-Gel transition, and the transition temperature being dependent on the polypeptide solution’s concentration. As shown in Figure 2A, the Sol-Gel transition temperature decreases with the increase of peptide concentration. The polypeptide solution with a concentration of 8 wt% exists in the form of low viscosity fluid, and has undergone a rapid Sol-Gel transition at about 29°C, which is more suitable for *in vivo* use. These two characteristics reflect the great potential as injection materials. The phase diagram trend of Gel-MP (Figure 2B) is basically consistent with that of Gel in Figure 2A, adding MP to polypeptide solution reduces the Gelation temperature of Gel, which may be related to the enhanced interaction between Gel and MP (Figure 2B). In addition to the Sol-Gel precipitation transition temperature, we also evaluated the rheological changes of the peptide solution without/with MP (Figures 2C, D). The storage moduli (*G*′) and loss moduli (*G*″) both exhibited an increase as the temperature rose. In addition, the Sol-Gel phase transition temperature obtained from the intersection of the storage moduli (*G*′) and the loss moduli (*G*″) is consistent with the Gel temperature obtained by the vial inversion method. This further demonstrates the temperature-sensitive and injectable nature of the Gel.

SEM was employed to further investigate the microstructure of the obtained Gel and Gel-MP constructs. As shown in Figure 1C, there were interconnected pore structures in the polypeptide Gel, which allows the infiltration of interstitial fluid to accelerate the degradation of polypeptide skeleton, and is also conducive to the adhesion of MP. As shown in Figure 1D, it is observed that MP is uniformly dispersed on the surface and inside of Gel after being combined with Gel (Figure 1D). The size and shape of MP and Gel did not change after mixing. Stirring at 4°C for 3 days did not damage the three-dimensional (3D) structure of MP, which is beneficial for the hydrophobicity and stability of the PLGA matrix. The above results indicate that Gel-MP constructs are suitable as injection platforms for *in vivo* applications.

### 3.2 Degradation of Gel-MP construct and sequential drug delivery

**FIGURE 3:**
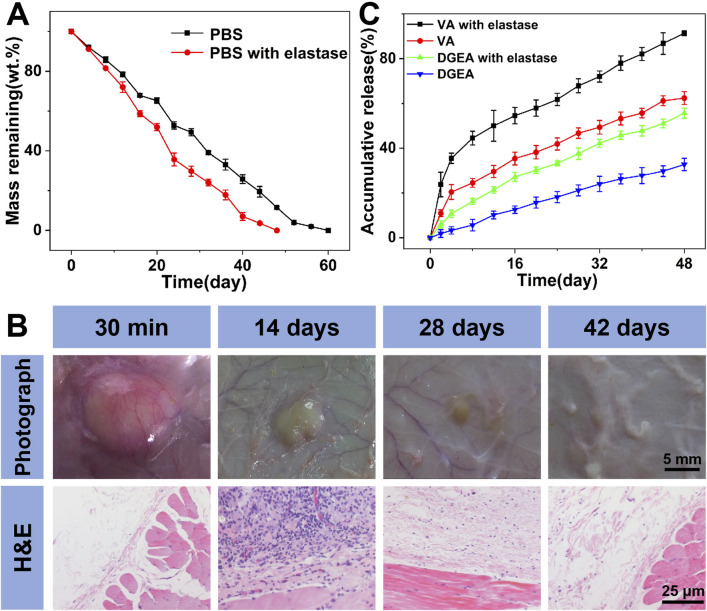
Degradation, biocompatibility, and drug release. **(A)** *In vitro* weight remaining profiles of Gel/VA-MP/DGEA incubated in PBS at pH 7.4 without or with elastase. **(B)** *In vivo* biodegradation and biocompatibility of Gel-MP construct; the Gel-MP solution was subcutaneously injected into mice. (Photographs around the implants were taken at 0 (30 min), 14, 28, and 42-day post-injection. H&E staining images around the implanted Gel after 0, 14, 28, and 42 days of subcutaneous injection of the Gel.) **(C)** *In vistro* VA and DGEA release from Gel/VA-MP/DGEA incubated in PBS without or with elastase. (Statistical data were represented as mean ± standard deviation (SD) (n = 3).

In order to observe the *in vitro* degradation of Gel-MP constructs, we conducted *in vitro* explanatory experiments on them in different culture media, namely, PBS and PBS containing 2.0 mg mL^−1^ elastase (Figure 3A). Compared to 60 days in the PBS group, the degradation rate of Gel-MP constructs increased in the presence of elastase and remained resistant for 50 days. At the same time, we also performed *in vivo* degradation assessments using rat models. As shown in Figure 3B, MP mixed Gel can be observed 30 min after subcutaneous injection, and gradually degraded within 42 days. The degradation rate *in vivo* is faster than *in vitro*, which may be due to various enzymes accelerating the degradation of Gel-MP constructs under the skin. In addition, the slightly acidic microenvironment caused by PLGA degradation is also due to accelerated degradation (Rocha et al., 2022; Yoo and Won, 2020; Su et al., 2021). Meanwhile, the biocompatibility of Gel-MP constructs was studied through H&E staining. As shown in Figure 3B, after 30 min of injection, although redness can be observed on the skin, minimal inflammation can be observed from H&E staining. After 7 days, based on the increase of inflammatory cells, the inflammation became severe. As the implant degrades, this phenomenon gradually alleviates. After 42 days, no inflammatory cells were observed in the tissue sections, indicating that the use of Gel-MP constructs as subcutaneous implants presents no safety concerns.

The *in vitro* release of VA and DGEA was carried out in PBS without and with elastase (Figure 3C). Gel/VA-MP/DGEA in PBS containing elastase showed an accelerated drug release rate due to the rapid degradation of Gel-MP constructs. The continuous release of DGEA ensures effective drug concentration *in situ* and avoids serious side effects. On the other hand, in the same period (i.e. 48 days), 91% of VA was released from Gel/VA-MP/DGEA, and a slight initial large release was observed in the first 8 days, attributed to the rapid diffusion of VA from the surface of Gel. VA is preferentially released from Gel to play an antibacterial role and reduce the inflammatory response associated with osteomyelitis, which also lays the foundation for the osteogenesis of the subsequently released DGEA and promotes the healing of bone defects.

### 3.3 Antibacterial sensitivity of Gel/VA-MP/DGEA

**FIGURE 4:**
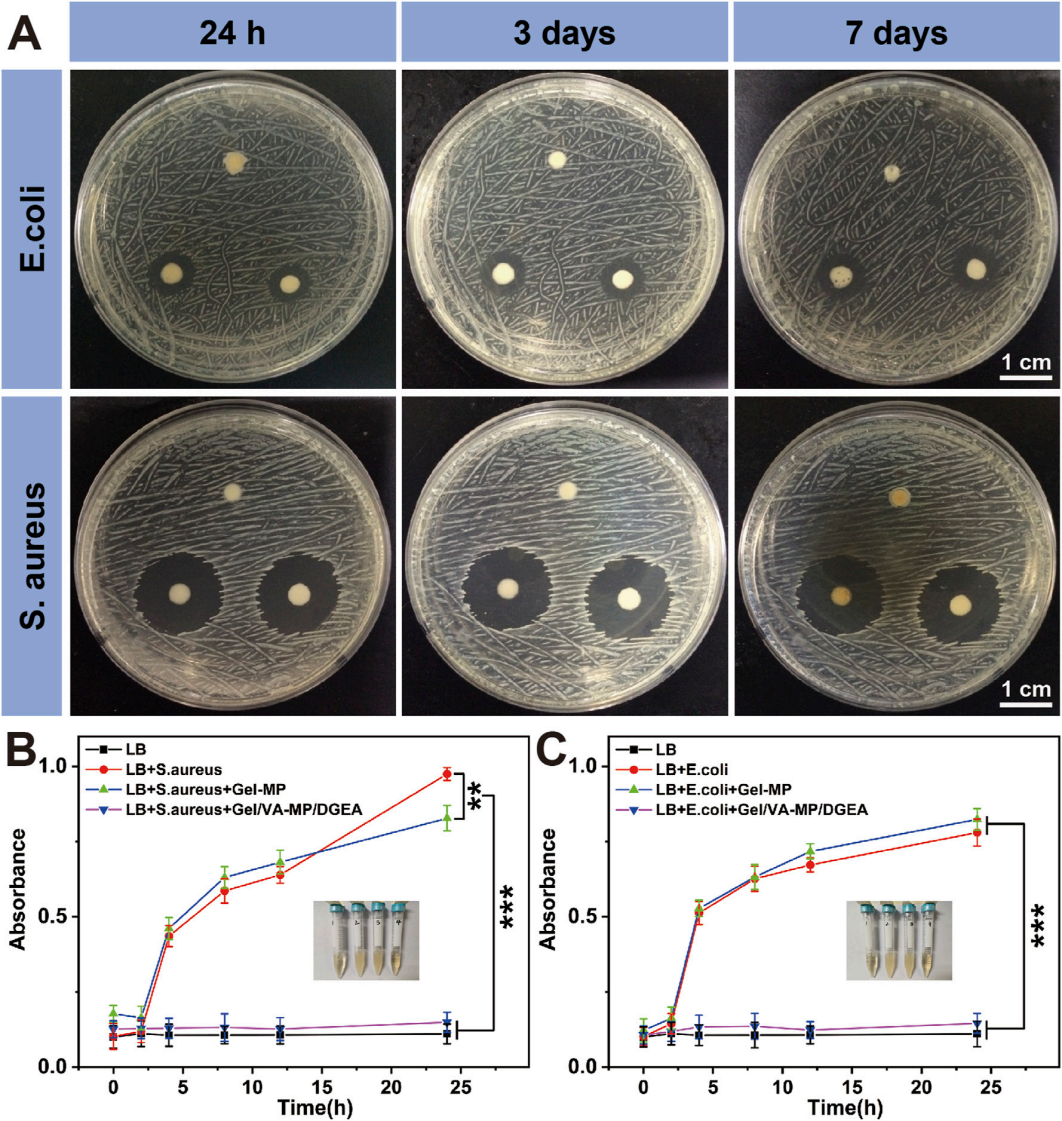
Antibacterial properties of Gel/VA-MP/DGEA. **(A)** The inhibition rings of *S. aureus* and *E. coli* with the zone inhibition assay, the clockwise group is Gel-MP, Gel/VA-MP, Gel/VA-MP/DGEA at 24 h, 3 and 7 days. Turbidity assay test tube photos of *S. aureus* **(B)** and *E. coli* **(C)**, from left to right are the LB, LB+ target bacteria, LB+ target bacteria+ Gel-MP, LB+ target bacteria+ Gel/VA-MP/DGEA. Changes in UV absorbance of **(B)** *S. aureus* and **(C)** *E. coli* in turbidity assay across different groups at 0, 2, 4, 8, 12, and 24 h. [Data were represented as mean ± SD (*n* = 3; **p < 0.01, ***p < 0.001)].

Because zone inhibition assay can be cultured in an incubator for a long time, up to a week, and is not easily contaminated by bacteria in the environment, it can demonstrate its antibacterial effect after 24 h of use. The zone inhibition assay as shown in Figure 4A, at the 24-h time point, the antibacterial ring of Gel-MP material in *E. coli* group was 6 mm, Gel/VA was 9.3 mm, and Gel/VA-MP/DGEA was 9.6 mm. The inhibitory ring of Gel-MP group in *S. aureus* culture dish is 6 mm, Gel-VA is 20.4 mm, and Gel-VA-MP/DGEA is 20.54 mm. From this comparison, when the material contains VA, it has a relatively good antibacterial effectiveness. In addition, the addition of MP/DGEA enhances the antibacterial ability of the material, which may be related to the accelerated release of VA after the addition of drug loaded microspheres. The results of cultures at 3 days and 7 days showed that although bacteria could grow normally on solid culture medium, the antibacterial ring did not shrink, indicating that Gel/VA-MP/DGEA material sustains antibacterial effects for more than 7 days. The diameter of the antibacterial ring of the drug loaded material in *S. aureus* culture dish is larger than that in *E. coli* culture dish, indicating that *S. aureus* exhibits greater sensitivity to VA than *E. coli*, and *S. aureus* is the most common pathogenic bacterium in chronic osteomyelitis. Therefore, the composite material holds promise for delivering an efficacious antibacterial action in the treatment of osteomyelitis (Zhang P. et al., 2023).

If the turbidity assay time is too long, the bacteria will overgrow and the nutrients in the culture medium will be insufficient. Therefore, it is used to demonstrate the antibacterial effect within 24 h. Figures 4B, C shows the turbidity assay result of *S. aureus* and *E. coli* at 0, 2, 4, 8, 12, and 24 h. It can be seen that whether it is for *S. aureus* or *E. coli* bacteria, Gel-MP showed significant antibacterial effects upon the addition of VA (*p* < 0.001). The LB group is a blank control group, without adding any materials or drugs. There was a significant difference in absorbance between the LB + bacterial group and the LB + bacterial + Gel-MP group and the blank control group, indicating that Gel-MP did not have a significant antibacterial effect. The LB + bacteria + Gel/VA-MP/DGEA group and Gel-MP group containing VA showed significant antibacterial effects with statistical significance (*p* < 0.001). The use of VA in clinical practice is increasing day by day, at present, VA has become the last line of defense for the treatment of MRSA and other drug-resistant bacteria in clinical practice (Guo et al., 2020). In this study, loading VA into Gel-MP not only ensures the minimum usage of VA, but also gradually exerts antibacterial effects as Gel-MP degrades over time, thereby creating a better microenvironment and timeframe for bone regeneration.

### 3.4 Ideal antibacterial and bone regeneration properties *in vivo*

**FIGURE 5:**
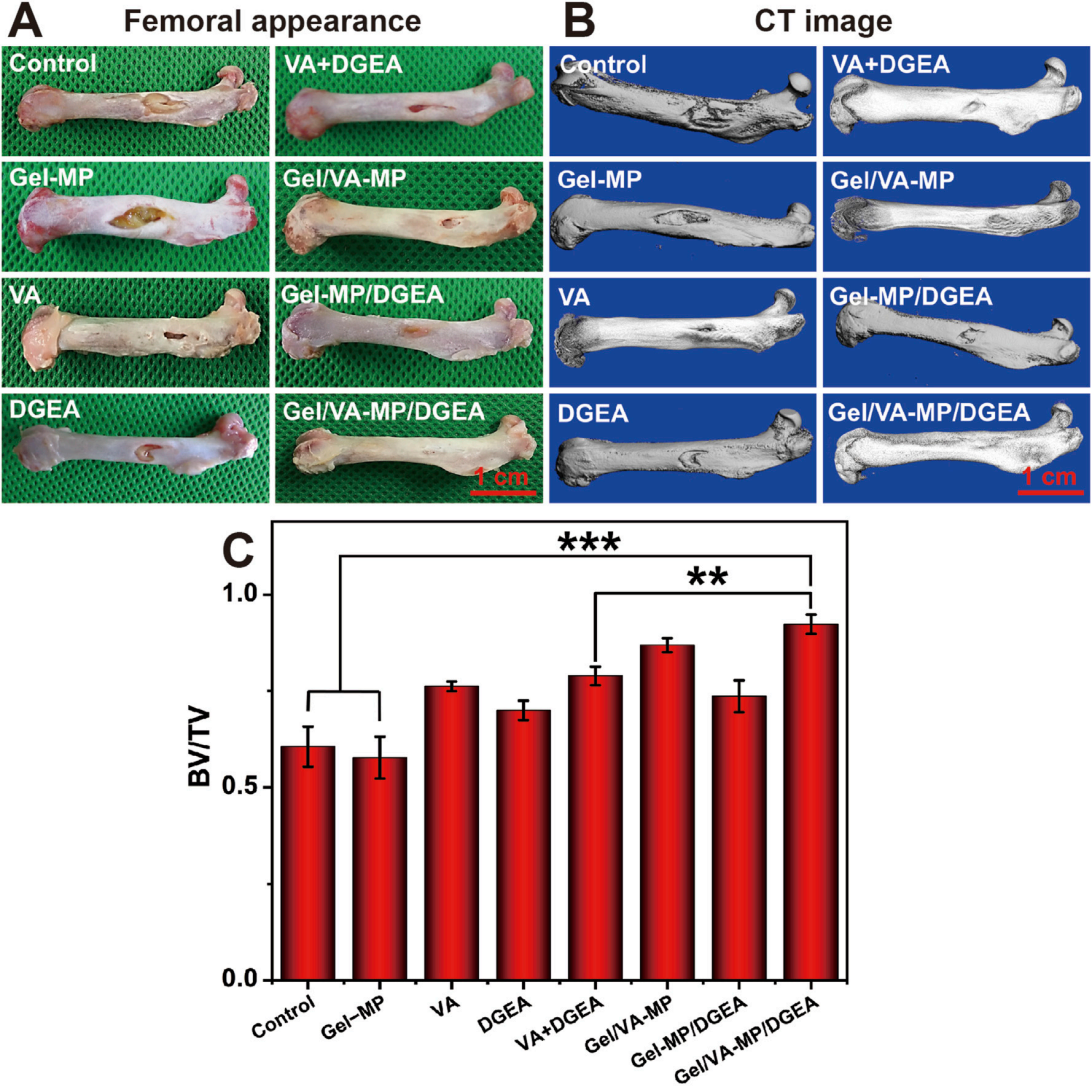
**(A)** Femoral appearance of rats for 4 months. **(B)** CT image of rats for 4 months. Scale: 5 mm. **(C)** BV/TV at 4-month post-treatment. (**p < 0.01, ***p < 0.001).

On the rat model of osteomyelitis, we evaluated the synergistic effect of VA and DGEA in the Gel-MP system. After modeling osteomyelitis in rats, there was extensive bone destruction in the femur and disordered arrangement of bone trabeculae. One week later, skin ulcers and pus leakage appeared on the wound surface. According to the external appearance of the femur (Figure 5A), it can be seen that the control group and Gel-MP group gradually worsened osteomyelitis after debridement, and the Gel-MP group exhibited larger femoral defects, a large amount of necrotic bone, and pus leakage. It is speculated that it may be related to the degradation of hydrogel, which will lead to the decrease of pH (Machado et al., 2022),and the microenvironment infected with bone environment is considered to be slightly acidic (pH 5.5–6.7) (Cicuéndez et al., 2018; Chen et al., 2018), which may be the result of metabolites produced by microorganisms. The VA group, DGEA group, VA + DGEA group, Gel/VA-MP group, and Gel-MP/DGEA group have limited bone defects with concavity and poor continuity of the marginal cortex, but the amount of new bone formation is significantly different from the control group and Gel-MP group, indicating a demonstrable therapeutic effect. The Gel/VA-MP/DGEA group showed the best bone repair effect visible to the naked eye. After 4 months, only gap sized defects were retained in the bone window, and there was some thin cortex at the gap, and the bone defect had been basically filled.

To more accurately assess bone defect repair, Micro-CT (Figure 5B) showed that compared with the control group and Gel-MP group, the VA group, VA + DGEA group had a lower depth of bone defects and better bone formation in the bone density, but the cortical defect site increased and the continuity of the marginal cortex was poor. The Gel-MP/DGEA group showed significant repair of bone density defects, with only cortical defects present. The Gel/VA-MP/DGEA group achieved the optimal treatment outcome, where the repair of the original fenestration defect site was basically completed.

To quantitatively analyze the repair of bone defects, we selected BV/TV (Figure 5C) as the indicator for evaluating bone regeneration. The BV/TV of the control group and Gel-MP group was less than 60%, while the BV/TV of the other drug loaded groups showed a significant enhancement compared to both the control group and the Gel-MP group. Compared with the injection of VA and DGEA alone, the combination of VA and DGEA also has a therapeutic effect on chronic osteomyelitis, with BV/TV reaching 78.9%. It is worth noting that the therapeutic effect of free VA is better than that of free DGEA, potentially attributable to free VA can bring more sustained antibacterial effects. Although DGEA can reduce the activation of M1 macrophages and prevent the occurrence of chronic inflammation (Jha and Moore, 2022), its efficacy still falls short of that achieved with VA. With the formation of bacterial biofilm, it will hinder the penetration of nutrients and antimicrobial agents, thereby affecting the osteogenic effect of DGEA (Conterno and Turchi, 2013). Therefore, the antibacterial and osteogenic effects have a significant sequential effect. The Gel/VA-MP/DGEA group exhibited the superior therapeutic outcome, and the BV/TV ratio reached 92%. Compared with the VA + DGEA group without drug loaded water Gel/microspheres composite system, there was still a significant difference (*p* < 0.01). This was due to the local sequential and continuous release of the two drugs (Zheng et al., 2020), which enabled it to have a more reasonable amount of new bone formation, indicating that the slow release drug loaded composite system could improve the degree of bone repair (Wang et al., 2022).

**FIGURE 6:**
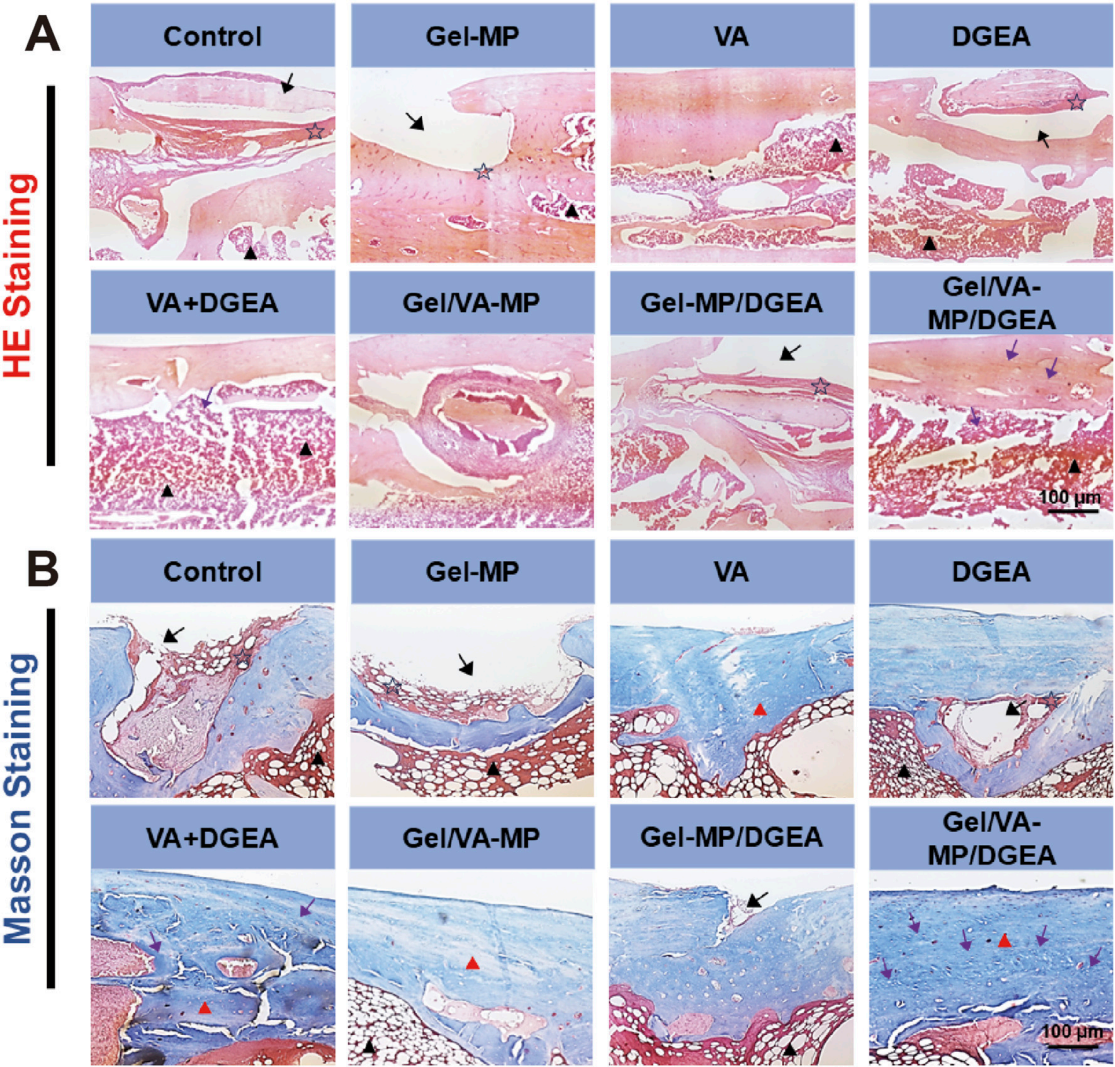
The **(A)** the HE staining **(B)** the Masson staining for Control, Gel-MP, VA, DGEA, VA + DGEA, Gel/VA-MP, Gel-MP/DGEA, Gel/VA-MP/DGEA with 4 months. Scale: 100 μm. Note: inflammatory cells (pentagram), bone marrow (black triangle), bone defect (black arrow), new bone tissue (purple arrow), collagen (red triangle).

HE staining (Figure 6A) showed that after 4 months of *S. aureus* infection, the Control group had a large number of inflammatory cell aggregates, while the Gel-MP group showed extensive bone defects with a large number of neutrophils, macrophages, and lymphocytes, and the generation of dead bone was visible at the edges. The inflammatory response was significantly reduced in the VA + DGEA group, Gel/VA-MP group, and Gel/VA-MP/DGEA group. In the Gel/VA-MP/DGEA group, a large amount of new bone formation was observed, the integrity of the bone marrow morphology was intact, and the defect window area was basically repaired. The composite drug loaded material has good anti-inflammatory and bone repair effects. Chronic osteomyelitis can recur frequently, attributed to the imbalance between host defense and bacterial invasion. The lack of local immune response hinders the clearance of infectious factors. Local ischemia can prevent inflammatory cell infiltration and systemic administration of antibiotics is possible. Therefore, a biofilm is formed on the dead bone, which protects internal bacteria from the infiltration of antibiotics, host immune defense cells, and antibodies. The immobilization form of pathogens within biofilms reduces sensitivity to antibiotics by 10^3^ compared to sterile conditions (Walter et al., 2012) report. In clinical practice, once the host defense system is inhibited, this phenomenon can further trigger the recurrence of osteomyelitis. Therefore, if the bacterial biofilm is not cleared, it will hinder bone regeneration, and the clearance of bacterial biofilm is the priority condition for the treatment of all chronic osteomyelitis. Although DGEA is a potent M1 macrophage inhibitor that can suppress inflammation, it will not have a good bone formation promoting effect if the bacterial biofilm is not cleared. Therefore, the application of DGEA in the treatment of chronic osteomyelitis plays a complementary role, and cannot be used as a standalone treatment method. With the removal and inhibition of biofilms, the use of DGEA drugs can promote bone regeneration faster and fill bone defects.

Further Masson staining was performed on the femoral sections (Figure 6B), yielding results similar to HE staining. Extensive bone defects were still observed on the Control and Gel-MP group sections, with a large accumulation of neutrophils, macrophages, and lymphocytes. The fibrous tissue of collagen was loose, and there was less new bone tissue. Dead bone formation could be seen at the edges. The collagen fibers in the DGEA and Gel-MP/DGEA groups are relatively loose and irregular, with evidence of inflammatory cell infiltration. In the VA + DGEA group and Gel/VA-MP/DGEA group, a large number of bone pits were observed, and after treatment with the sustained-release composite drug loaded materials, it was observed that the defect window area had been basically repaired, with new bone formation and dense and regular collagen fiber tissue. Therefore, the composite drug loading material Gel/VA-MP/DGEA has a good effect in promoting the generation of collagen, and the filled collagen provides good flexibility for bone (Yu and Wei, 2021).

**FIGURE 7:**
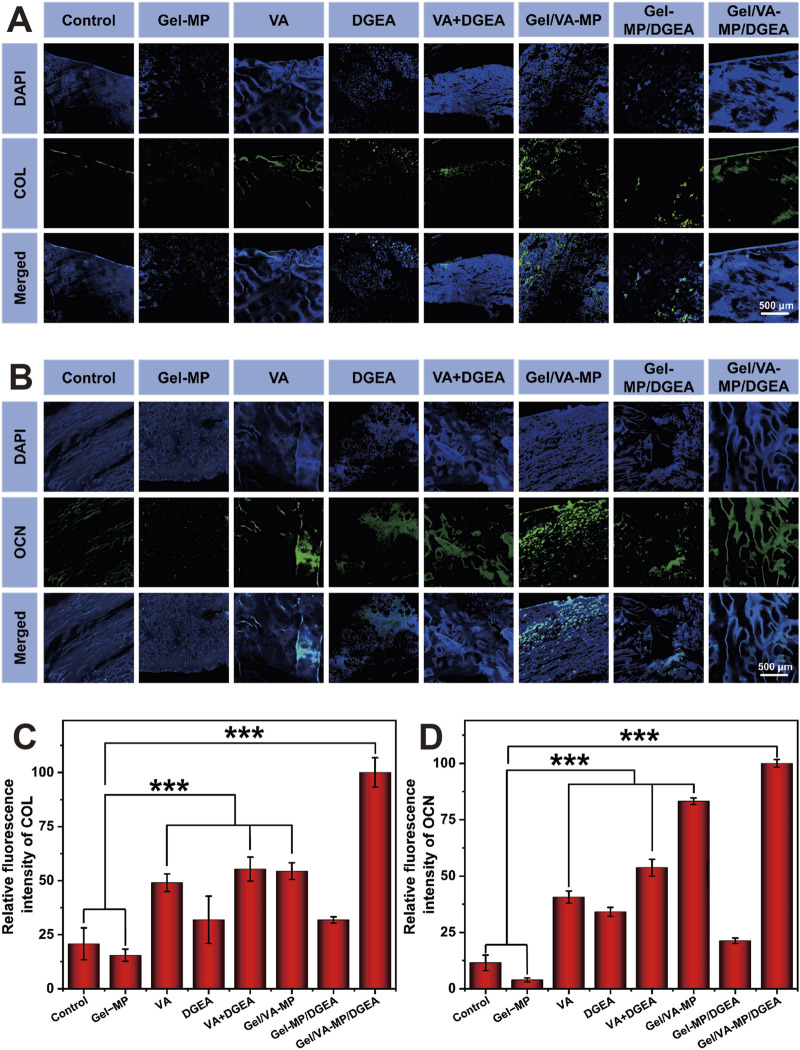
Qualitative and semi quantitative immunofluorescence of COL and OCN. **(A)** COL immunofluorescence nuclear antigen staining labeled with Cy5.5. **(B)** Cy5.5 labeled OCN immunofluorescence nuclear antigen staining. Semi quantitative analysis of immunofluorescence staining: **(C)** relative fluorescence intensity of COL; **(D)** The relative fluorescence intensity of OCN. (Statistical data is expressed as mean ± standard deviation (n = 3). ***p < 0.001).

To further confirm the therapeutic effect of the Gel/VA-MP/DGEA group, *in vitro* immunohistochemical testing was performed. Analyze the osteogenic effect by using the levels of type I collagen (COL I) and osteocalcin (OCN). COL is the most abundant protein in bone matrix, while OCN is one of the specific proteins of osteoblasts and the most abundant non collagen protein in bone (Li et al., 2016; Vijayalekha et al., 2023). Here, we employed Cy5.5 labeled COL and OCN for cell staining. Figures 7A, B show that in the Gel/VA-MP/DGEA composite drug loading group, the green fluorescence region is the most abundant. However, although there are also active regions of bone collagen and osteoblasts in the Control group and Gel-MP group, the stained area is significantly smaller and the tissue morphology is irregular.

Using ImageJ software for semi quantitative analysis (Figures 7C, D), it was found that the fluorescence intensity and fluorescence area of the control group were much lower than those of the other groups, indicating that only a very small number of osteoblasts appeared in the defect area. The COL expression level of the Control group was only 15.5% of that of the Gel/VA-MP/DGEA composite loading group, and the OCN expression level was only 4.0%. The COL expression level of the Gel-MP group was only 20.8% of that of the Gel/VA-MP/DGEA composite loading group, and the OCN expression level was only 11.5%. The VA group, VA + DGEA group, and Gel/VA-MP group equipped with VA also showed ideal bone activity, with COL expression levels more than three times that of the Control group and Blank group. The OCN expression level in the Gel/VA-MP group surpassed eight times that of the blank group. The fluorescence intensity of the Gel/VA-MP/DGEA composite drug loading group was significantly higher than that of all drug loading groups, indicating an effective increase in the number of osteoblasts and bone matrix content, with good osteogenic activity.

**FIGURE 8:**
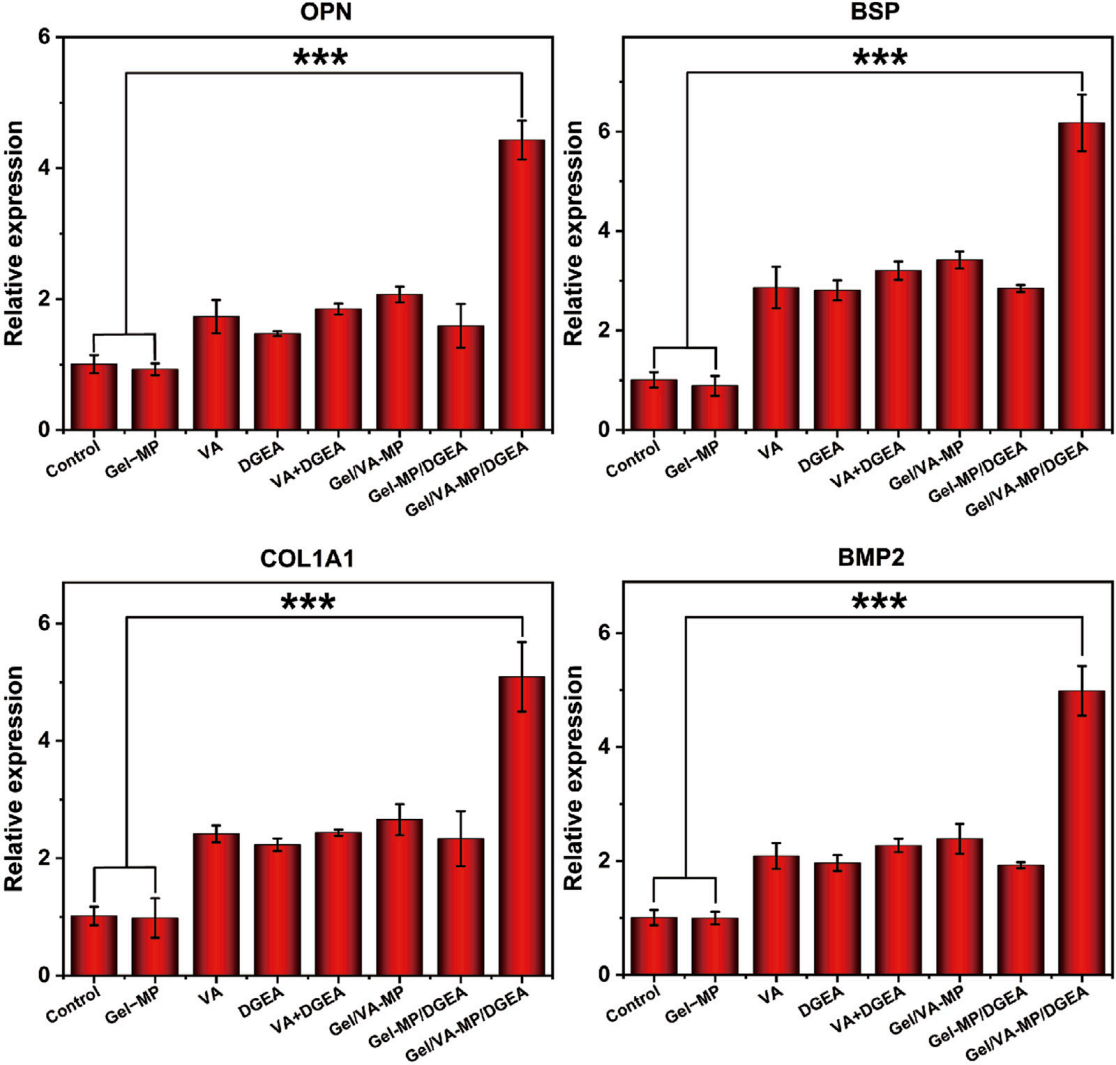
Gel/VA-MP/DGEA composite materials exhibit more ideal osteogenic differentiation. RT-PCR for the Gene expression of OPN, BSP, COL1A1 and BMP2 among the eight groups. n = 3. (***p < 0.001).

**FIGURE 9:**
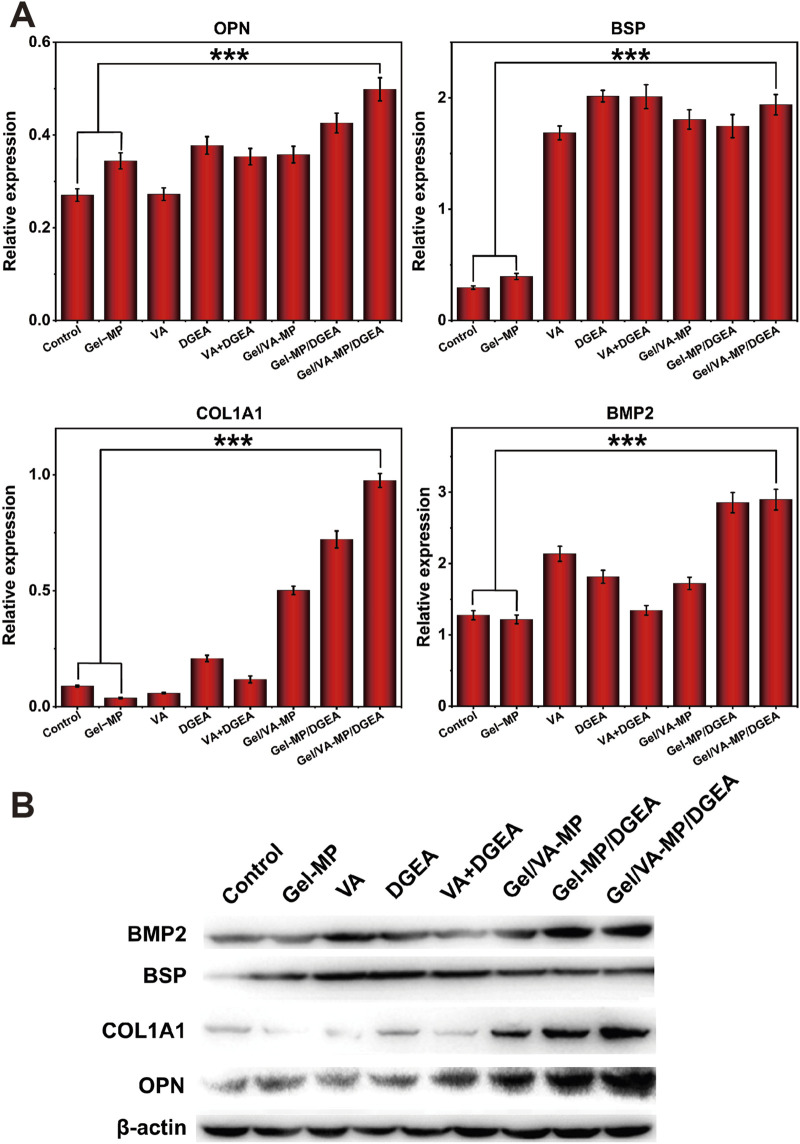
Gel/VA-MP/DGEA composite materials exhibit more ideal osteogenic differentiation. **(B)** Western blotting for the expression of OPN, BSP, COL1A1 and BMP2 among the eight groups, measured in the fourth month. **(A)** Corresponding quantitative analysis of protein expression in **(B)** n = 3. (***p < 0.001).

In order to further explore the osteogenic potential of the Gel/VA-MP/DGEA group at the molecular level, we conducted RT-PCR and Western blot experiments. Detect the expression of osteogenic related genes and proteins after 4 months. From Figure 8, the relative expression levels of osteogenic genes (OPN, BSP, COL1A1, BMP2) in the composite drug loading group were significantly higher than those in the other groups, more than twice that of the single drug loading group Gel/VA-MP and Gel-MP/DGEA, and approximately 4–5 times higher than in the control group. The relative expression levels of osteogenic genes (OPN, BSP, COL1A1, BMP2) in VA, DGEA, VA + DGEA, Gel/VA-MP, Gel-MP/DGEA in other drug loaded groups were not significantly different, but there was also a good improvement compared to the control group. In Western blot experiments (Figures 9A, B), it was found that the results were similar to the osteogenic related genes observed in RT-PCR experiments. Through the electrophoretic analysis of OPN, BSP, COL1A1, and BMP2 related proteins, comparing the groups, and conducting quantitative grayscale analysis, the levels of various osteogenic proteins in the newly formed tissue at the defect site treated with the Gel/VA-MP/DGEA composite were notably elevated compared to both the Control group and the Gel-MP group.

Bone regeneration is based on the stimulation of various signaling pathways to promote the proliferation and differentiation of MSCs to generate new bone tissue, which is further mineralized and matured. These changes are closely relateSd to the expression level of genes (Yang and Liu, 2021). OPN, also known as “bone bridge” protein, can interact with various cell surface receptors, such as the RGD sequence. The RGD sequence is abundant in the bone tissue matrix and can mediate important extracellular matrix and intercellular interactions (Gravallese, 2003). OPN plays an important role in the mineralization of bone tissue, promoting the formation of crystal nuclei (Zhang M. et al., 2023), which aligns with the observed upregulation in expression levels during the experimental studies. BSP protein and its mRNA can be detected in mature osteoblasts, but not in precursor cells (Kriegel et al., 2022). Therefore, we chose BSP to evaluate bone maturity. The expression of BSP in the Gel/VA-MP/DGEA group was significantly higher than that in other groups, indicating that the newly formed bone tissue in the defect has begun to mineralize and mature, transforming into mature bone tissue. As is well known, compared to fibrous connective tissue, bone tissue is notably enriched in Type I collagen fibers (Li et al., 2021), and the expression level of COL1A1 in the composite drug loading group was found to be significantly higher compared to other groups. The BMP2 gene has been shown to play an important role in bone development, facilitating the differentiation of osteoblasts and bone formation. It is almost involved in all stages of bone regeneration, and stimuli from other pathways, which is mediated by BMP, can also trigger osteogenic responses (Arrabal et al., 2013). The increased expression of osteogenic related proteins (OPN, BSP, COL1A1, BMP2) demonstrated that the composite drug loaded Gel/VA-MP/DGEA group can indeed promote bone regeneration, increase the volume of new bone tissue in the defect area, accelerates bone tissue mineralization, and facilitates the transformation into mature bone tissue.

### 3.5 Security of Gel/VA-MP/DGEA *in vivo*

Safety is one of the most important factors in the potential clinical application of any advanced chemotherapy drug formulation. In this study, the safety of all formulations was determined by detecting changes in histopathological morphology of visceral organ slices.

**FIGURE 10:**
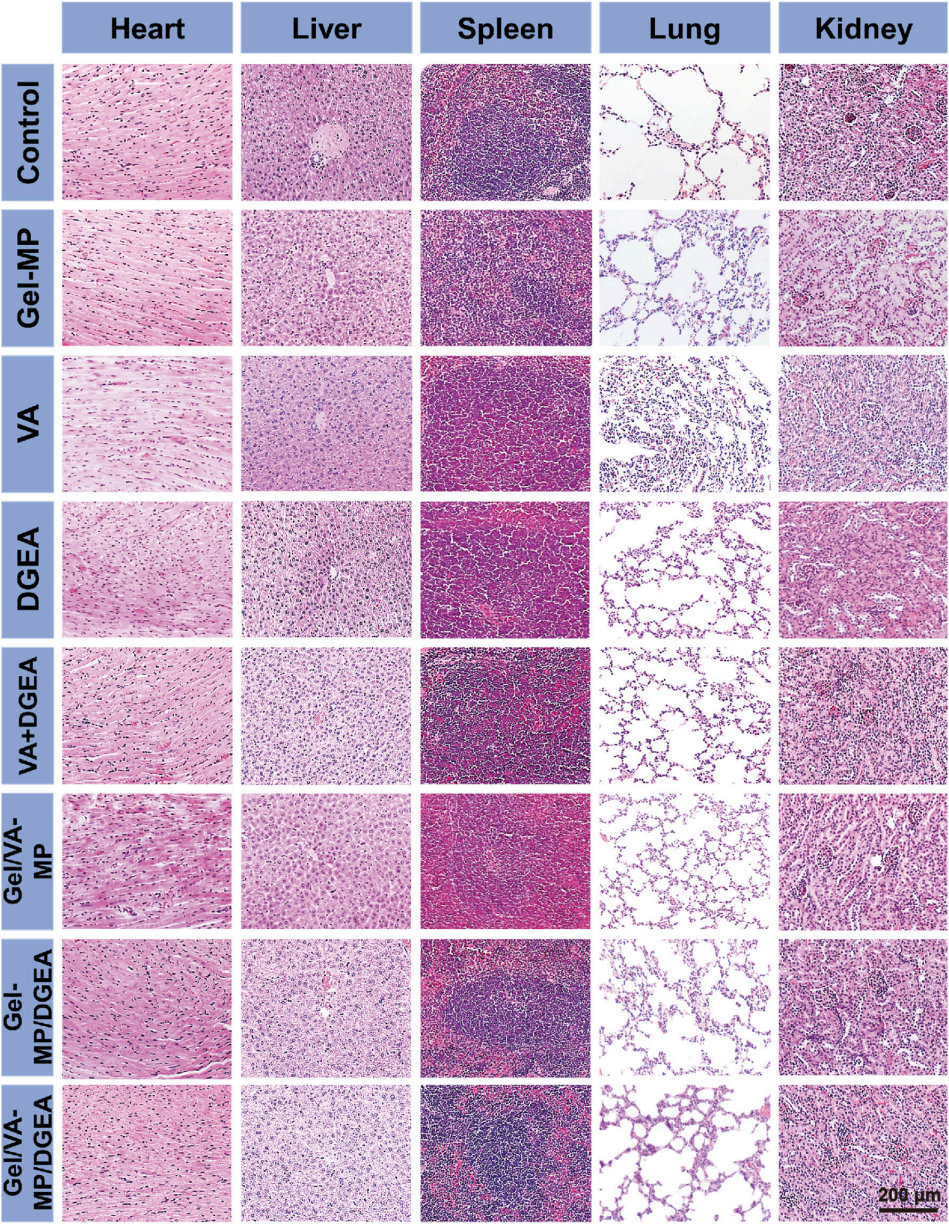
Security *in vivo*. *Ex vivo* histological analyses of main organ sections after treatment with PBS as a control, Gel-MP, VA, DGEA, VA + DGEA, Gel/VA-MP, Gel-MP/DGEA, or Gel/VA-MP/DGEA.

To evaluate the safety of all test formulations, histopathological analysis was performed on major organs (such as the heart, liver, spleen, lungs, and kidneys) by using H&E staining after all treatments (Figure 10). Observe the histological morphology of each organ. It can be seen that there are no significant abnormalities in the pathological sections of important organs such as the heart, liver, spleen, lungs, and kidneys in each group. The results indicate that the Gel-MP construct is non-toxic to human organs, and neither Gel-MP carrying VA nor MP carrying DGEA will cause toxic reactions to important organs. This experiment shows that the Gel-MP construct does not cause damage to important organs and has good clinical application prospects.

## 4 Conclusions

Injectable Gel-MP structures were prepared using PLAF-*b*-PEG-*b*-PLAF and PLGA as the skeletons of Gel and MP, respectively, for continuous administration. After subcutaneous injection, the Gel-MP carrier completely degraded within 42 days and exhibited good biocompatibility. In terms of *in vivo* application, after injection the site of osteomyelitis defects, VA is initially released from the Gel/VA-MP/DGEA system, effectively exerting its antibacterial activity and controlling infection. Then, as Gel degrades, DGEA is released from MP to induce bone formation, resulting in the effect of filling bone defects. These findings suggest that Gel/VA-MP/DGEA has good anti-inflammatory and osteogenic effects *in vivo*, and has high potential in the treatment of femoral osteomyelitis.

## Data Availability

The original contributions presented in the study are included in the article/Supplementary Material, further inquiries can be directed to the corresponding authors.
