# Supplementary material for: Treatment of chronic osteomyelitis with gradient release of DGEA and vancomycin hydrogel-microsphere system and its mechanism
Source: Front Pharmacol. 2024 Nov 11;15:1499742. doi: 10.3389/fphar.2024.1499742 (PMC11586164; doi:10.3389/fphar.2024.1499742)
Supplement: Supplementary file 1 [file DataSheet1.docx]

Treatment of Chronic Osteomyelitis with Gradient Release of DGEA and Vancomycin Hydrogel−Microsphere System and its Mechanism

**MATERIALS AND METHODS**

**1.Materials.**Poly(ethylene glycol) (PEG, number average molecular weight (*M*_n_) =4000 g mol^−1^) was purchased from Sigma-Aldrich (Shanghai, P. R. China). L-Alanine N-carboxyanhydride (L-Ala NCA) and L-phenylalanine N-carboxyanhydride (L-Phe NCA) were obtained from Shanghai Yeexin Biochem&Tech Co., Ltd. (Shanghai, P. R. China). PLGA (Intrinsic viscosity (*η*) = 0.6 dL g−1, LA:GA = 75:25, mol/mol) was obtained from Changchun Sino Biomaterials Co., Ltd. (Changchun, P. R. China). DGEA was bought from GL Biochem (Shanghai) Ltd. Poly (vinyl alcohol) (PVA), sodium hydroxide (NaOH), and sodium dodecyl sulfonate (SDS) were purchased from Shanghai Chemical Reagent Co., Ltd. (Shanghai, P. R. China). VA was purchased from Shanghai yuanye Bio Technology Co., Ltd. Elastase was ordered from Aladdin Reagent Co., Ltd. (Shanghai, P. R. China).

**2.Synthesis and Characterization of PLAF-*b*-PEG-*b*-PLAF.** The copolymer of

poly(L-alanine-co-L-phenylalanine)-block-poly(ethyleneglycol)-co-poly(L-alanine-co-L-phenylalanine) (PLAF-b-PEG-b-PLAF) was synthesized through the ring-opening polymerization (ROP) of L-Ala NCA and L-Phe NCA using NH2-PEG-NH2 as a macroinitiator, as depicted in supplementary Scheme S1. Firstly, 5.0 g of NH2-PEG-NH2 was azeotropically distilled with toluene at 120 °C S-3 to remove trace of water. Subsequently, 2.3 g of L-Ala NCA and 0.4 g of L-Phe NCA dissolved in 100.0 mL of N, N-dimethylformamide (DMF) were added. After 3 days of reaction at room temperature, the obtained solution was poured into 500.0 mL of ethylether, and the precipitate was collected. The final product was dried under vacuum with the yield of 87.6%. Proton nuclear magnetic resonance (1H NMR) spectrum was performed on a 300 MHz Bruker spectrometer (Bruker Scientific Instruments, Billerica, MA, USA) with trifluoroacetic acid-d (TFA-d) as a solvent to confirm the successful synthesis of the copolymer.

**3.Preparation of Blank and DGEA-Loaded Microspheres (MPs).** Water-in-oil-in-water (W_1_/O/W_2_) double emulsion technology was used to prepare the blank and DGEA-entrapped PLGA MP (MP/DGEA). For the preparation of MP/DGEA, 135.0 mg of PLGA and 24.0 mg of Tween-80 were firstly dissolved in 4.5 mL of dichloromethane containing 15.0 mg of DGEA. After the complete dissolution of PLGA and DGEA, the above mixture was injected in 50.0 mL of PVA aqueous solution with the mass fraction of 1.0 wt.%. After emulsification for 3 min by the high-speed shearing machine at a rotating speed of 3000 r/min, the emulsion was poured into 100.0 mL of double distilled water and then stirred for 6 h. After full volatilization of dichloromethane, products were centrifuged and collected at 2000 r/min for 3 min, followed by washing with distilled water for 3 times. MP/DGEA was obtained as white powder after lyophilization. The blank MP was prepared according to the similar procedure without DGEA.

**4.Drug-Loading Content and Efficiency of MP.** Sodium hydroxide−sodium dodecylsulfonate (NaOH−SDS) method was used to measure the drug-loading content (DLC) and drug-loading efficiency (DLE) of MP/DGEA. 2.0 mg of MP/DGEA was dispersed into 1.0 mL of NaOH−SDS (5 wt.%, NaOH 0.1 mol L^−1^) solution and placed in the oscillation box (HZQ-X100, Donglian Co., Ltd., Harbin, P. R. China) for overnight shocking at constant temperature of 37 °C. After the decomposition of MP/DGEA, the clear supernatant liquid was taken via centrifugation, and the DGEA concentration in the supernatant was measured.

$DLC\left( \% \right)=\frac{W_{loaded drug}\left( mg \right)}{W_{loaded MP}\left( mg \right)}\times100\%$ （1）

Where, W_loaded drug_ and W_loaded MP_ were denoted as the weight of loaded drug and MP/DGEA, respectively.

$DLE\left( \% \right)=\frac{W_{loaded drug}\left( mg \right)}{W_{feeding drug}\left( mg \right)}\times100\%$ （2）

Where, W_loaded drug_ and W_feeding drug_ were denoted as the weights of loaded drug in

MP/DGEA and the feeding drug in the course of drug encapsulation, respectively.

**5. Morphologies of MP and Gels.** PLAF-*b*-PEG-*b*-PLAF without or with 5.0 wt.%

MP were dissolved in phosphate-buffered saline (PBS) with a concentration of 8.0

wt.%. After full dissolution, 0.5 mL of solution was transferred into a vial and placed in

the thermostat at 37 °C for 30 min to form Gels. Next, the Gels were frozen in liquid

nitrogen and lyophilized. The morphologies of MP and freeze-dried Gel piece were

observed by scanning electron microscope (SEM; Philips XL30, Eindhoven, The

Netherlands).

**6. Phase Diagram and Gelation Time.** The sol−gel transition behavior of polypeptide Gel without or with MP in PBS was evaluated by a vial inverting test with a temperature increase of 1.0 °C per 5 min. The diameter of vial is 11 mm. First, the polypeptide solutions at different concentrations of 6.0, 7.0, 8.0, and 9.0 wt.% without or with 5.0 wt.% MP were stirred at 4 °C. After dissolution, 0.5 mL of the above solution was drawn into a vial. The gelation was defined as that no flow of the system was observed within 30 s when the vial was inverted.

**7. Rheology Analyses.** The rheological study of polypeptide solution in PBS with the optimized concentration of 8.0 wt.% without or with 5.0 wt.% MP was tested on a MCR 302 rheometer (Anton Paar, Graz, Austria). The test temperature was set to increase from 10 to 70 °C at a speed of 0.5 °C min^−1^. The diameter of the plate was 25 mm, and the gap was set at 0.5 mm. The storage moduli (G′s) and loss moduli (G′′s) of Gels were detected with the parameters of strain and frequency at 1% and 1 Hz, respectively. The rheological study of polypeptide solution in PBS with 5.0 wt.% MP/DGEA and 0.5 wt.% VA was tested in the same way above.

**8. In Vitro Release Profiles of MP/DGEA.** 30.0 mg of MP/DGEA powder and 1.0 mL of PBS without or with 2.0 mg mL−1 of elastase were added to 2.0 mL centrifuge tube. Subsequently, the tube was placed into the oscillation at 37 °C with continuous shaking at 70 rpm. The upper buffers were collected after centrifugation at 1000 r min−1 and replaced by fresh buffer, and the remained MP/DGEA was placed into the oscillation again every day. The concentrations of DGEA in the reserved release solutions were measured by high-performance liquid chromatography (HPLC; Waters S-6 1525 system with a Waters C18 column and a Waters 2489 ultraviolet/visible (UV/vis) detector, Waters, Milford, MA, USA). Acetonitrile−water (60:40, *V/V*) was used as elution with a flow rate of 1.0 mL min^−1^ . The absorption wavelength (λ_abs_) was set at 230 nm, and Breeze software was used for datum analyses. The peak of DGEA appeared at 6 min.

**9. Statistical Analyses.** All the statistical data were given as mean ± standard deviation (SD). The statistical differences were analyzed according to the paired Student's *t*-test. Statistical significance and highly statistical significance were presented when the *P* value was less than 0.05, and 0.01 and 0.001, respectively.


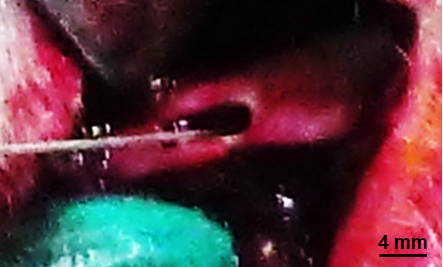


**Figure S1.** Injecting S. aureus into bone defects.


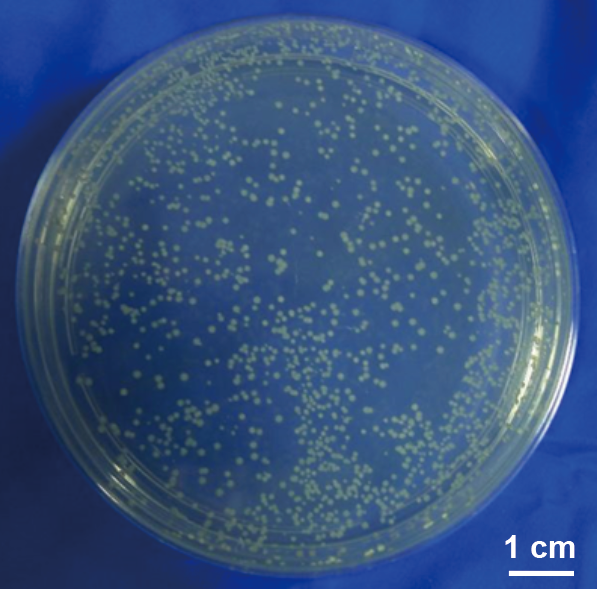


**Figure S2.** Bacterial culture of bone marrow cavity blood at the site of modeling after completion of modeling.


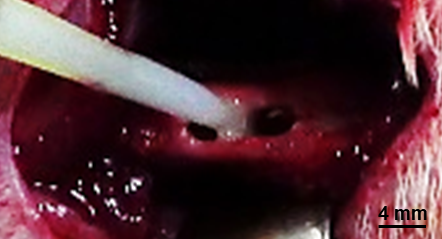


**Figure S3.** Inject Gel into the bone defect to evaluate the effect of subsequent osteomyelitis treatment.


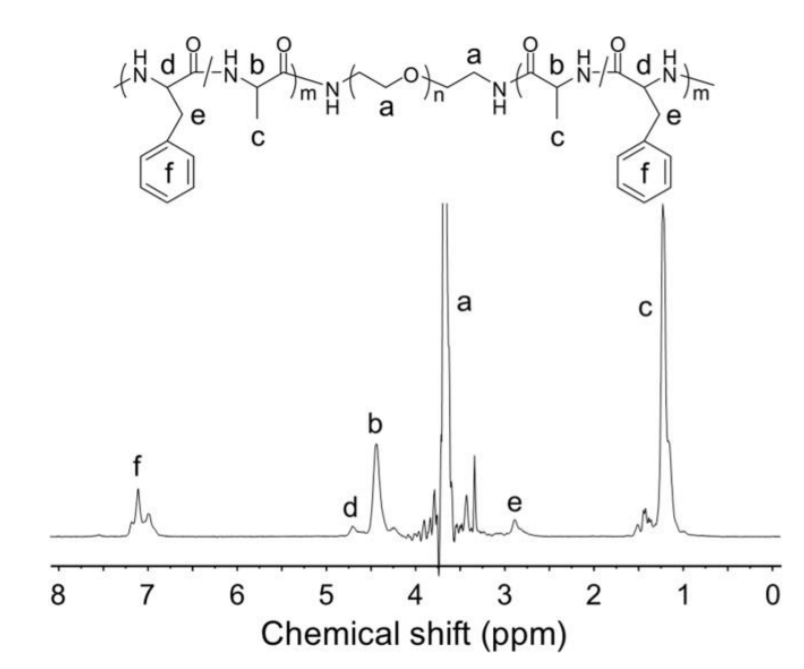


**Figure S4.** ^1^H NMR spectrum of PLAF-*b*-PEG-*b*-PLAF.


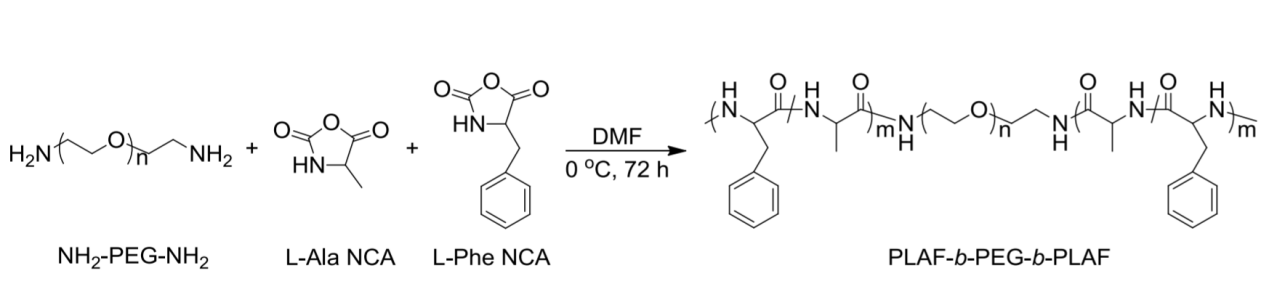


**Scheme S1.** Synthetic route of PLAF-*b*-PEG-*b*-PLAF
